# The impact of changes in taste, smell, and eating behavior in children with cancer undergoing chemotherapy: A qualitative study

**DOI:** 10.3389/fnut.2022.984101

**Published:** 2022-09-30

**Authors:** Mirjam van den Brink, Minke M. ter Hedde, Emmy van den Heuvel, Wim J. E. Tissing, Remco C. Havermans

**Affiliations:** ^1^Laboratory of Behavioral Gastronomy, Centre for Healthy Eating and Food Innovation, Maastricht University Campus Venlo, Venlo, Netherlands; ^2^Princess Máxima Center for Pediatric Oncology, Utrecht, Netherlands; ^3^Department of Pediatric Oncology and Hematology, University of Groningen, University Medical Center Groningen, Groningen, Netherlands

**Keywords:** taste, smell, eating behavior, quality of life, childhood cancer

## Abstract

**Background and aims:**

Taste changes are the third most common bothersome symptom during treatment in children with cancer. However, it is still unclear what the essence of these taste changes are, to what degree concomitant changes in sense of smell qualify this bothersome treatment symptom and how much of an impact these changes have on the life of children with cancer. The aim of this study was to explore characteristics of both taste and smell changes and to gain insight into the impact of these changes in children with cancer receiving chemotherapy.

**Methods:**

Semi-structured interviews were performed until data saturation was achieved in each age group (6–12, 13–17 years). This resulted in an in-depth description of taste and smell changes, including its impact on the life of 27 children with various cancer types receiving chemotherapy. Thematic analysis of interview data was performed.

**Results:**

Interview data could be grouped into three main themes, namely changes in (1) taste, (2) smell, and (3) eating behavior. As expected, most children reported experiencing taste and smell changes just after start of treatment, but changes varied greatly between children; that is, some reported changes in intensity (increased or decreased), whereas others reported different perceptions or preferences (from sweet to savory). Taste and smell changes (regardless of direction) negatively impacted quality of life, with these changes commonly described as “disappointing” or “frustrating.” Interestingly, particular chemotherapeutic agents were frequently mentioned regarding taste and smell changes, prompting sensory-specific coping strategies. Children's eating behavior changed in terms of alterations in food liking and appetite, sometimes due to chemosensory changes, but children also mentioned specific medication or hospital food being responsible for their altered eating behavior.

**Conclusions:**

Both taste and smell changes are common in children with cancer. The essence of these changes varies widely, but taste and smell changes are generally considered bothersome treatment symptoms. Ways to cope with taste or smell changes specifically were described by the children warranting further research and offering the opportunity for enhancing patient-centered care.

## Introduction

Children with cancer receiving chemotherapy often experience bothersome symptoms, such as nausea and pain. Taste changes have been found to be the third most common bothersome symptom during treatment, reported by 60.3% of the children (1). Several studies indicate that taste changes are indeed common in children with cancer (2–4). However, experienced changes in taste do not reflect a change in taste function per se. Decreased smell function (anosmia/hyposmia) for instance, is often mistaken for loss of taste function in the general population (5). Colloquially, taste refers to a multisensory percept (flavor); that is, the integrated chemosensory experience of gustatory, olfactory, and somesthetic stimulation (6). So, smell and taste, and their combined perception of flavor, are all important characteristics of food that determine liking and preferences and play a distinct but related role in eating behavior (7). However, little is known whether smell changes also occur in children with cancer undergoing treatment, and if so, to what extent.

When exploring chemotherapy-induced chemosensory changes in adult cancer patients, a systematic review suggests that there is insufficient evidence that chemotherapy influences taste in a uniform manner when focusing on sensitivity and intensity of taste qualities (8). However, it seems that the changes in liking for foods and other aspects of flavor perception have the greatest influence on the perception of food during chemotherapy. This is confirmed by a qualitative study among adults with cancer, showing that patients experience a range of symptoms which they identify as “taste” problems during chemotherapy which in fact mostly relate to the broader phenomena of flavor and hedonics (9). Among children with cancer, taste changes have been previously described in a heterogeneous way, mostly referring to hedonics (food tasting “different” or “bad”) (10). In addition, flavors could be experienced blander or more extreme in children receiving cancer treatments. However, it is still unclear what exactly a child with cancer means when it talks about “taste” problems. Does it reflect a shift in taste function? Does a possible change in taste function lead to a concomitant shift in food preferences? Is the sense of smell affected? If so, does that play a likely role in food enjoyment and the degree of perceived taste changes? Until now, these aspects are understudied as most studies solely focus on taste changes in children with cancer. For that reason, changes in smell and other aspects potentially influencing eating behavior should be explored as well, because a complete overview of chemosensory problems might facilitate the development of effective strategies to manage these changes.

It should be noted that taste and smell changes associated with anti-cancer treatments (notably chemotherapy) affect food intake and nutritional status in adult patients (11, 12). In addition to that, chemosensory changes seriously impact adult patients' daily life and wellbeing (9, 13). This appears to be just as true in the case of childhood cancer. In a recent study, adolescents with cancer (12–18 years) most frequently reported cancer-specific health-related quality of life (HRQoL) problems related to chemosensory changes such as “food not tasting good” (54.3%) and “nausea caused by food and smells” (61.4%) (14). Again, it is unclear what qualities define the essence of these food-related changes experienced by children with cancer. At the moment, we are still working on a longitudinal study in which we quantitatively measure taste and smell function in children with cancer. However, if we measure taste and smell changes it is largely unclear what makes these changes bothersome and what impact they have on the daily lives of so many children undergoing chemotherapy. These are meaningful questions as qualifying children's experiences with taste and smell changes during treatment offers the opportunity to enhance patient-centered care. Therefore, we interviewed children with cancer, as part of the longitudinal study, asking them about their experiences with changes in taste and/or smell while receiving chemotherapy and how these changes impact(ed) their daily lives.

## Materials and methods

### Study design

This is a qualitative study to explore experiences with and the impact of taste and smell changes in children with cancer undergoing chemotherapy, using semi-structured interviews. Interviews were held between January and September 2021.

### Participants and recruitment

This study was performed at the Princess Máxima Center for Pediatric Oncology in Utrecht, the Netherlands. All children newly diagnosed with cancer, consecutively admitted to the Princess Máxima Center, were asked to participate in a prospective cohort study called SENSORY-2, including several quantitative measurements of taste and smell function during treatment. Children in the SENSORY-2 study needed to be between 6 and 17 years old, diagnosed with a hematological, solid, or brain malignancy, and currently treated with chemotherapy.

As quantitative measurements alone do not inform us on all aspects concerning children's chemosensory disturbances, all children within SENSORY-2 were invited to participate in the current study comprising a semi-structured interview. At the time of the interview, these children had already undergone chemotherapy for at least 3 months so that they were sufficiently able to talk about their potential experiences with taste and smell changes. Children were not purposefully selected on reporting changes in taste and smell function (convenience sampling) and interviews were held with the children who were first enrolled in SENSORY-2 (pending data saturation).

### Data collection

MB carried out the semi-structured interviews. We planned to enroll at least 10 children in each age group (6–12, 13–17 years). After interviewing ~20 participants, we decided that data saturation was achieved if no new information was obtained from the three subsequent interviews in each age group, resulting in a total of 27 interviewees. Interviews were held during (day) admission at the Princess Máxima Center and lasted between 10 and 27 min. During the interviews, children were often accompanied by a parent who was allowed to participate in the conversation, preferably at the end of the interview to make any additions. The interview guide was developed in collaboration with a pediatric oncologist, nutritional scientist/dietitian, health scientist, representative of the patient organization, and two psychologists and was based on key topics from literature and experiences from a previous study (4). Interviews covered descriptions of changes in taste, smell, or preferences, timing of these changes, its association with specific treatments or interventions, the impact (practical, social) on daily life, and strategies to handle these changes. Interviews were audio recorded.

The youngest children (6–12 years) were invited to use the ‘write and draw technique' (15). School-age children are familiar with drawings and writings and this technique is therefore considered a child-friendly method to collect data from children. Children who wanted to use this technique, were given paper and pencils and were asked to draw or write: (1) their favorite foods; (2) foods that taste differently; and (3) smells that have changed or became unpleasant since treatment with chemotherapy. Afterwards, children were asked to talk about their drawings and writings, followed by further questions about their experiences.

### Data analysis

Data were analyzed using thematic analysis, a qualitative method for identifying, analyzing and reporting themes (16, 17). Thematic analysis was chosen to provide a rich description of the data. All digitally recorded interviews were transcribed verbatim and imported as text documents in ATLAS.ti (Scientific Software Development GmbH, Berlin, Germany), a qualitative analysis program. MB and MH initially coded all transcripts (open coding), compared coding, and resolved discrepancies. RH then reread all coded transcripts and supplemented feedback to create a coding manual that was finalized after feedback from EH. Afterwards, all transcripts were coded again using the final coding manual. Themes were derived from the data in an inductive way.

The team had regular discussions throughout the duration of the project. Further, MB presented findings and tentative conclusions for further discussion with other experts at a research meeting. These discussions were conducted with the intent to avoid that our conclusions being particular to the perspective of just one researcher. However, it should be noted that several members of the team (MB, WT, RH) have previously investigated taste and smell changes in children with cancer, which may have influenced the interviewing and coding of the data.

### Ethical consideration

All procedures performed in this study were in accordance with the ethical standards of the institutional and/or national research committee and with the 1964 Helsinki Declaration and its later amendments or comparable ethical standards. The Medical Ethics Review Committee of the University Medical Center Utrecht (UMCU), the Netherlands, approved this study as subpart of the SENSORY-2 study (METC N19.809). Written informed consent was obtained from the parents, and from children ≥12 years. A 15-point checklist of criteria for good thematic analysis was used for reporting and writing (17).

## Results

Twenty-seven children with cancer were included in this study. Participant demographics and clinical characteristics can be found in Table 1. During the interviews, the majority of children reported both taste and smell changes (*n* = 20, 74.1%), whereas only one girl did not report any changes in taste or smell perception. When focusing on taste and smell separately, it can be noticed that taste changes (*n* = 23, 85.2%) and smell changes (*n* = 22, 81.5%) are frequently reported since the start of treatment.

**Table 1:** Characteristics of the included childhood cancer patients.

**Characteristics**	**Patients (*n* = 27)**
**Sex**, female (*n*, %)	14 (51.9)
**Age** (median, range)	12 (6–17)
6–12 years (*n*, %)	14 (51.9)
13–17 years (*n*, %)	13 (48.1)
**Diagnosis**	
Hematologic malignancy (*n*, %)	23 (85.2)
Brain tumor (*n*, %)	1 (3.7)
Solid tumor (*n*, %)	3 (11.1)
**Months since diagnosis** (median, range)	3.7 (2.3–5.3)
**Taste changes since treatment** (*n*, %)	23 (85.2)
**Smell changes since treatment** (*n*, %)	22 (81.5)
**Both taste and smell changes since treatment** (*n*, %)	20 (74.1)
**Nausea in last 2 days** (*n*, %)	6 (22.2)
**Mucositis in last 2 days** (*n*, %)	1 (3.7)

Thematic analysis identified three overarching main themes, namely (1) changes in taste; (2) changes in smell, and (3) altered eating behavior. In discussing changes in taste and smell, interview data could be grouped into five sub-themes: *nature, timing, association, impact, and coping*. Data regarding altered eating behavior was grouped into two sub-themes: *food liking and appetite* (Table 2). Each theme and sub-theme are explored using illustrative quotes from the children to illuminate findings.

**Table 2:** Themes.

**(1) Taste changes**	**(2) Smell changes**	**(3) Altered eating behavior**
I. **Nature** •Intensity •Quality •Bad taste •Preference	I.**Nature** •Intensity •Quality •Hedonics	I. **Food liking** •Less desired foods •More desired foods •Home-cooked meals •Fluctuation of food choices
II. **Timing** •Onset •Transience	II. **Timing** •Onset •Transience	II. **Appetite** •Changes in appetite •Corticosteroids
III. **Association** •Mucositis •Specific chemotherapeutic agent •Treatment-related intervention	III. **Association** •Nausea •Specific chemotherapeutic agent •Treatment-related intervention •Negative experience	
IV. **Impact** •Frustration •Dissapointment	IV. **Impact** •Irritation	
V. **Coping** •Adding (strong) flavors •Trying foods •Brushing teeth •Eating sweets •Drinking	V.**Coping** •Avoiding unpleasant smells •Covering unpleasant smells	

### Taste changes

#### Nature

To understand how children describe possible taste changes, we first asked them about their definition of taste. This was described in a uniform way, namely: how something (food or drink) tastes, how strong it tastes, and whether it is tasty or not.

Then we continued the conversation about taste changes during chemotherapy. Children described a variety of taste changes, most often expressed by alterations in intensity, quality, preferences, or the presence of a bad taste. Taste perception in terms of intensity could be either increased or decreased:

“*Everything tastes much stronger” (girl, 11 years)*

“*My taste was then completely gone, I couldn't taste anything” (boy, 17 years)*

Children also mentioned that some foods tasted very different (quality) than before chemotherapy or than they were used to. Others experienced a bad taste in their mouth:

“*Sometimes it has a completely different flavor, for example my father always makes soup on Sundays and suddenly the zucchini soup tasted like chicken soup or something. Not really a recognizable soup” (girl, 11 years)*

“*I often have a very bad taste in my mouth the whole day long and it doesn't matter what I eat or drink, that taste just stays the whole time” (boy, 16 years)*

A majority of the children noticed a change in their taste preference, with sweet being less preferred and a remarkable preference for salty or savory foods:

“*I would now rather eat something savory than something sweet, that is a really big change” (boy, 16 years)*

“*When I had chemotherapy, I leaned much more towards salty. I really liked chips, or those salty pretzels” (girl, 17 years)*

#### Timing

In general, taste changes were experienced since the start—or in the first month—of treatment:

“*When I had chemo for the first time” (girl, 9 years)*

The course of these changes was relatively heterogeneous with some children describing that these changes are mainly present in the first week after a cycle of chemotherapy and then gradually normalizes, while others describing taste changes as being continuously present since the start of treatment:

“*I think that after 1 or 2 days you already notice it and that lasts for about the first week. Then you really notice it, your tongue also feels a bit numb. Then after a week and a half you may have it [normal sense of taste] back, but then you have half a week to taste your food and then you have a new course [of chemotherapy]” (boy, 17 years)*

#### Association

When it comes to treatment-related symptoms associated with taste changes, oral mucositis was mentioned by some of the children being responsible for their altered taste perception:

“*My mucous membranes were completely damaged. The taste change is because of that” (boy, 15 years)*

In addition, specific chemotherapeutic agents (methotrexate) or chemotherapy cycles (e.g., OEPA, consisting of vincristine, doxorubicin, prednisone, and etoposide) were mentioned regarding a decreased perception of taste intensity. Furthermore, dexamethasone (a corticosteroid) was mentioned by several children in relation to changes in taste preferences:

“*During the first two OEPA courses I experienced it [change in taste] badly. My taste was then really just gone” (boy, 17 years)*

“*That was very strange, he only wanted savory when he was on the dexa[methasone]” (mother of 6-year old boy)*

Moreover, a lot of children notice a flavor or retronasal smell during saline flushing of their central venous line which was reported to be like “glue,” “medicine” or “salty”:

“*A salty, uh yes… it's really a kind of smell that you taste in your mouth. It's really odd” (girl, 14 years)*

#### Impact

Taste changes negatively influence daily lives of most children, with these changes commonly described as “frustrating” or “disappointing”:

“*It was extremely frustrating. I smelled yummy food everywhere, but when it ended up in my mouth, I didn't like it. It was just really annoying to go through that, it makes you not want to eat” (girl, 17 years)*

“*I really noticed that I couldn't really enjoy my food because those flavors didn't come out properly. I didn't like that at the time either” (girl, 14 years)*

#### Coping

Children tried several things to manage changes in taste. Coping strategies included adding (strong) flavors or trying (new) foods when suffering from any form of changes in taste:

“*If we have chips, I'll have sweet chili sauce with them, and I'll dip the chips in it. That has a very strong flavor which I could taste” (girl, 13 years)*

“*I try to eat some things more often to get used to the new taste” (girl, 11 years)*

A bad taste in the mouth, especially when not eating, was resolved by brushing teeth, eating sweets, or drinking something:

“*I just tried rinsing my mouth and brushing my teeth quite often, but nothing helped at that point” (girl, 17 years)*

“*When I eat lollipops, I don't taste the bad taste” (boy, 17 years)*

### Smell changes

#### Nature

Smell changes were most often expressed by alterations in intensity, quality, or hedonics. Similar to taste, smell perception could be either increased or decreased in intensity:

“*Well I burned a scented candle recently but that also smelled too strong” (girl, 14 years)*

“*My nose doesn't smell things far away but does smell things close to my nose” (boy, 6 years)*

Sometimes, smells were perceived differently compared to before chemotherapy:

“*I remember how that perfume smelled in he past, it was always a bit sweet but now it just smells very different, a bit like sweat” (boy, 12 years)*

A majority of the children indicated that they perceived unpleasant smells since the start of treatment, mainly concerning food odors, body odors, and hospital odors:

“*Dinner just stinks, but nothing else” (boy, 17 years)*

“*Your breath stinks; she never said that before” (mother of 17-year old girl)*

“*At one point I had troubles with the smell of that disinfectant stuff. The smell was just continuously present” (girl, 17 years*)

#### Timing

Changes in smell intensity—increased or decreased—were most often noticed since the start of treatment. However, the perception of unpleasant smells, especially food odors and hospital odors, was only experienced after several visits to the hospital. From that moment on, these odors were continuously experienced as unpleasant, while, for example, a decreased smell intensity was mainly experienced in the 2 weeks after a cycle of chemotherapy and then slowly recovered:

“*In the first two weeks, I also had a lot of smell loss. It also took a long time to come back, maybe two weeks” (boy, 17 years)*

“*At the beginning of treatment she said she didn't like it (smell of alcohol) very much, but then it wasn't so dominant. I think it started in the middle of treatment, after 6 or 7 cycles” (mother of 10-year old girl)*

#### Association

Although a majority of the children also experienced taste alterations besides changes in smell, these concepts were rarely linked to each other. However, nausea was often mentioned in relation to smell. On the one hand, children indicated that unpleasant odors cause nausea, but on the other hand, it was also noted that smells are poorly tolerated when you already feel nauseous:

“*On a normal day it's okay if I smell fries or something, but on a day when I'm nauseous I think: go away” (girl, 17 years)*

“*The smell of food makes me nauseous and usually makes me vomit” (girl, 11 years)*

In addition to nausea, the use of corticosteroids (dexamethasone and prednisone) was mentioned a few times in relation to increased smell sensitivity or perception of body odors:

“*What struck me is that whenever she had prednisone or dexa[methasone], if I had had coffee she would say: Gross, your breath stinks” (mother of 12-year old girl)*

Moreover, a lot of children stated not liking the smell of ethanol on their central venous catheter patch, which is generally only experienced by themselves:

“*When they change that PICC line patch, I can smell it for a few days afterwards but mommy doesn't smell it” (girl, 14 years)*

Odorants are frequently linked to specific experiences or locations. In this case, children with cancer described that certain smells, for example from soup or hand sanitizer, directly remind them of the hospital which is unpleasant

“*They often eat soup here [at the hospital], and then I smell, you know, the scent of soup. And then when I'm home and I smell tomato soup, I start to think about the hospital. It just makes me nauseous” (boy, 17 years)*

#### Impact

Changes in smell, in whatever form they appear, are mostly experienced as “irritation.” Children also describe unpleasant food and hospital odors being continuously present, which has a negative impact on their mood:

“*It's kind of an irritation. You want to get rid of that smell. It follows you and that's not fun, it's not nice and it has to go” (girl, 17 years)*

“*It's a bit of an issue because she starts to breathe really strange and nothing helps which makes her unhappy. It makes her a bit grumpy” (mother of 10-year old girl)*

#### Coping

Children generally described coping strategies for managing unpleasant smells, rather than solutions for a decreased or increased smell sensitivity. Coping strategies include avoiding and covering of unpleasant smells:

*“I often say at dinner time: give me only potatoes with Greek yogurt. I just can't tolerate the smell of other things being cooked” (girl, 17 years)*

“*I usually ran upstairs around dinner time because it smells so bad but I can still smell it [food being cooked] upstairs. Then I spray deo[dorant], that suddenly smells very nice” (girl, 11 years)*

### Altered eating behavior

#### Food liking

Children commonly described a decrease in food liking for certain foods, such as vegetables and chocolate. Sometimes, this reduction in food desire was linked to perceived changes in taste or attributed to the smell of that particular food, but this was not always clear:

“*Well I really think: disgusting. I don't feel like that. Chocolate, yuck, no. While I normally think: oh yum, chocolate” (girl, 17 years)*

On the other hand, fruits and fatty snacks, such as chips, fries, and noodles, were often referred to as more desirable foods. A lot of children also explicitly stated that they prefer home-cooked meals:

“*I started eating noodles more often” (girl, 14 years)*

“*Well, I really don't like the hospital food, I just like our own food” (girl, 14 years)*

Additionally, desired foods seem to fluctuate during treatment as mentioned by the following participant:

“*Sometimes I like fruit, then candy and then, a few days later, I like something else and then candy doesn't taste good anymore” (boy, 17 years)*

#### Appetite

Children noticed their appetite changed since the start of treatment, with some children experiencing a decreased appetite and others having an increased appetite. Particularly dexamethasone and prednisone were mentioned when it comes to an insatiable appetite and binge eating:

“*I have the feeling that the signal between her head and tummy is just turned off” (mother of 8-year old girl)*

“*During the first two courses I ate a lot, a lot more. But that's because of the prednisone, you're much hungrier then” (boy, 17 years)*

## Discussion

This qualitative study provided an in-depth exploration of how children with cancer experience changes in taste and smell, but also eating behavior, and the impact these changes have on their daily lives. As expected, most children reported experiencing taste and smell changes right after the start of treatment, but changes varied greatly between children; that is, some reported changes in intensity (increased or decreased), whereas others reported different perceptions or preferences. Taste and smell changes often affect the daily lives of children with cancer, with these changes commonly described as “disappointing,” “frustrating” or “annoying.” Interestingly, particular chemotherapeutic agents (e.g., methotrexate, corticosteroids) were frequently mentioned regarding taste and smell changes, prompting sensory-specific coping strategies. Children's eating behavior changed in terms of alterations in food liking and appetite, sometimes due to chemosensory changes, but children also mentioned specific medication or hospital food being responsible for their altered eating behavior.

In our study, taste changes were heterogeneously experienced and described by children with cancer. This is in accordance with a previous qualitative study, in which pediatric patients noted that food tasted “different” or “not right,” but could also be experienced in terms of food tasting “bland” while others experienced more extreme flavors (10). Like that study, taste changes were described to start with treatment initiation and coping strategies, such as sucking on candy, brushing teeth, and modifying food choices, were mentioned by our patients as well. Although children with cancer try to resolve taste changes, the current study emphasizes that these changes seriously reduce food enjoyment and affect daily life in various ways. In contrast to the study from Loves et al., we also investigated changes in smell which have been shown to have a major impact on the eating experience of children with cancer as well. Further research should focus on how children and parents want to be supported on this topic. Interestingly, a recent feasibility study investigating nutrition education and cooking workshops for families of children with cancer, showed that the workshop “changes in taste during cancer therapy” was most useful to parents (18). This underscores how much difficulty parents and children have with taste changes and that providing ways to deal with these changes is very valuable.

A shift from sweet to savory foods was a common finding among children in our study. Previous qualitative reports describe a similar pattern among children with cancer undergoing chemotherapy, in which children repeatedly indicated that they avoid chocolate and candy since the start of treatment (3, 10, 19). The mechanism for such a change in taste preference (from sweet to savory) is not clear. A comparative analysis exploring taste perception and food behavior among adults undergoing chemotherapy showed that a reduced sweet sensitivity is indeed most common, consequently affecting food intake (20). The authors suggest that sweet taste receptors might be more susceptible to chemotherapy or that neural responses to sweet ligands are altered. In contrast, increased sweet taste sensitivities have also been found in breast cancer patients and children with cancer undergoing chemotherapy, which may also explain why sweet dishes appear to be less preferred during chemotherapy (4, 21). Further research is needed to verify these changes in sweet preference and/or sensitivity in cancer patients receiving chemotherapy.

A follow-up study from Loves et al. among 108 pediatric cancer patients suggested that optimizing chemotherapy-induced nausea and vomiting control, but also mucositis prevention, may reduce taste changes (22). Children in our study also associate nausea and mucositis with changes in smell and taste, respectively. Interestingly, nausea was explicitly mentioned in relation to smell. That is, smells from food might cause nausea or exacerbate existing nausea, which in turn leads to the child not wanting to eat. Adequate symptom management might help in the case of existing nausea, but might not be a solution for nausea induced by (food) odors that can appear at any time. The question however is, whether an intervention to control nausea improve taste changes (in terms of increased or decreased sensitivity) or rather prevent a child with cancer from conditioned taste aversions (CTA). It is well-known that nausea can reinforce a strong aversion for a food stimulus (its smell and/or taste), allowing children with cancer to develop a CTA (23). This too highlights the importance of exploring not only taste changes, but also smell sensitivity, during treatment with chemotherapy.

In general, extreme sensitivities to specific odorants such as perfume, cleaning solutions, food cooking, and hand sanitizer have been reported by adult cancer patients receiving cancer treatment (11). Children in our study gave similar examples of unpleasant smells they had experienced during treatment, but also frequently noted that they were much more likely to perceive their own body odor—or someone else's—than they were used to before chemotherapy. Further research should clarify if corticosteroids might cause these alterations or heightened sensitivity, as some children and parents indicated. The impact of smell changes is most apparent around meals. In contrast to adults, children with cancer did not talk about its social implications such as the inability to eat with family and friends (13, 24). However, they do use coping strategies such as the elimination of strong-smelling foods or eating in another room to deal with this unpleasant situation.

Applying thematic analysis as a method to explore the experiences of children with cancer undergoing chemotherapy resulted in a rich description of food-related changes and its impact on children's daily lives. This study also includes a broad range of experiences, as we did not purposefully select participants who reported smell or taste changes before. Furthermore, this is the first study that explores children's experiences with changes in smell during treatment with chemotherapy. Like taste changes, changes in smell also have a major impact on the eating experience and social lives of children with cancer, which requires more attention in the future. Moreover, a relatively large number of children participated in this study at the same time point in their treatment. However, our findings must be interpreted in the light of several study limitations. Similar to adults, children in the current study frequently confused the concepts of taste and smell. This is the result of inductive analysis where we wanted to have an open view on how children themselves describe their chemosensory disorders. However, explaining the definitions of taste and smell could have facilitated the interpretation of the results. Moreover, childhood cancer represents a heterogeneous set of diseases among children of various ages. Nevertheless, the majority of our participants were children with hematological malignancies, which may have consequences for drawing general conclusions. In addition to this qualitative study, changes in taste and smell need further investigation through quantitative measurements, including its consequences regarding nutritional status and quality of life.

## Conclusions

In conclusion, our findings show that both taste and smell changes are highly prevalent and diverse in children with cancer receiving chemotherapy, but are generally considered bothersome treatment symptoms. Ways to cope with these changes were extensively described, including adding (strong) flavors or covering unpleasant smells for taste and smell changes, respectively. Future research should explore ways to manage taste and smell changes in children with cancer undergoing chemotherapy. In the meantime, current findings can be used to improve patient-centered care.

## Data availability statement

The original contributions presented in the study are included in the article/Supplementary materials, further inquiries can be directed to the corresponding author.

## Ethics statement

The studies involving human participants were reviewed and approved by the Medical Ethics Review Committee of the University Medical Center Utrecht (UMCU), the Netherlands. Written informed consent was obtained from the parents, and from children 12 years and under.

## Author contributions

MB conceptualized and designed the study, collected data, reviewed data, participated in interpretation of the data, drafted the initial manuscript, and revised the manuscript. MH reviewed data, participated in interpretation of the data, and helped to draft the manuscript. EH reviewed data, participated in interpretation of the data, and critically reviewed the manuscript for important intellectual content. WT participated in study design, supervised its execution, helped with interpretation of the data, and critically reviewed the manuscript for important intellectual content. RH participated in study design, supervised its execution, reviewed data, helped with interpretation of the data, and critically reviewed the manuscript for important intellectual content. All authors read and approved the final manuscript.

## Funding

The Laboratory of Behavioral Gastronomy was supported by the Dutch Province of Limburg. The funding organization had no role in the design and conduct of the study; collection, management, analysis and interpretation of the data; preparation, review or approval of the manuscript or decision to submit the manuscript for publication.

## Conflict of interest

The authors declare that the research was conducted in the absence of any commercial or financial relationships that could be construed as a potential conflict of interest.

## Publisher's note

All claims expressed in this article are solely those of the authors and do not necessarily represent those of their affiliated organizations, or those of the publisher, the editors and the reviewers. Any product that may be evaluated in this article, or claim that may be made by its manufacturer, is not guaranteed or endorsed by the publisher.
