# Supplementary material for: The impact of changes in taste, smell, and eating behavior in children with cancer undergoing chemotherapy: A qualitative study
Source: Front Nutr. 2022 Sep 30;9:984101. doi: 10.3389/fnut.2022.984101 (PMC9565543; doi:10.3389/fnut.2022.984101)
Supplement: Supplementary file 1 [file Data_Sheet_1.pdf]

Interview guide SENSORY-2

Introduction (grand-tour question)
Think of your favorite foods. What would you like to eat at the moment?

Part 1: taste

- What do you think of when you hear the word "taste"?
- Have you noticed that certain foods taste differently or that your taste has changed with receiving chemotherapy?
  - Prompts: what exactly changed? (loss of taste, change of intensity or quality, or otherwise)
- Have you noticed that you now like certain foods more – or less?
  - Prompts: what do you like (or dislike) about it? How did it happen?
- Can you tell me when you first noticed this?
- Can you tell me when this is most noticeable or what makes it worse?
  - Prompts: before/during/after chemotherapy? Related to location (hospital/home setting)?
- Thinking of your changes in taste, how did these changes affect you?
  - Prompts: how do you notice those changes everyday? What do you find difficult/stupid/fun about it?
- Have you tried any strategies to make the taste changes less annoying or bothersome?
  - Prompts: did you change your diet? If so, what foods do you eat more/less?
- Do you ever have a (bad) taste in your mouth even when you are not eating?
  - Prompts: how would you describe this taste?

Part 2: smell

- Have you noticed that your smell has changed with receiving chemotherapy?
  - Prompts: what exactly has changed? (loss of smell, change of intensity or quality, hedonics/aversions, or otherwise)
- Can you tell me when you first noticed this?
- Can you tell me when this is most noticeable or what makes it worse?
  - Prompts: before/during/after chemotherapy? Related to location (hospital/home setting)?
- Thinking of your changes in smell, how did these changes affect you?
  - Prompts: how do you notice those changes every day? What do you find difficult/stupid/fun about it?
- Have you tried any strategies to make the smell changes less annoying or bothersome?

Closing questions

- Is there anything else about your smell and taste changes that you feel we didn't talk about?
- Do you have any advice for the Princess Máxima Center? For example, should we pay more attention to certain foods, (hospital) smells, et cetera?
